# Development of a Bestatin-SAHA Hybrid with Dual Inhibitory Activity against APN and HDAC

**DOI:** 10.3390/molecules25214991

**Published:** 2020-10-28

**Authors:** Jiangying Cao, Wei Zhao, Chunlong Zhao, Qian Liu, Shunda Li, Guozhen Zhang, C. James Chou, Yingjie Zhang

**Affiliations:** 1 Key Laboratory of Chemical Biology (Ministry of Education), School of Pharmaceutical Sciences, Department of Medicinal Chemistry, Cheeloo College of Medicine, Shandong University, 44 West Wenhua Road, Jinan 250012, China; 60030103@sdutcm.edu.cn (J.C.); zhaowei20200220@163.com (W.Z.); 201715682@mail.sdu.edu.cn (C.Z.); liuqian199805@163.com (Q.L.); m15133634175@163.com (S.L.); 17853102863@163.com (G.Z.); 2 School of Pharmacology, Shandong University of Traditional Chinese Medicine, Jinan 250355, China; 3 Department of Drug Discovery and Biomedical Sciences, South Carolina College of Pharmacy, Medical University of South Carolina, Charleston, SC 29425, USA

**Keywords:** hybrid, dual inhibitor, CD13, histone deacetylase, aminopeptidase

## Abstract

With five histone deacetylase (HDAC) inhibitors approved for cancer treatment, proteolysis-targeting chimeras (PROTACs) for degradation of HDAC are emerging as an alternative strategy for HDAC-targeted therapeutic intervention. Herein, three bestatin-based hydroxamic acids (**P1**, **P2** and **P3**) were designed, synthesized and biologically evaluated to see if they could work as HDAC degrader by recruiting cellular inhibitor of apoptosis protein 1 (cIAP1) E3 ubiquitin ligase. Among the three compounds, the bestatin-SAHA hybrid **P1** exhibited comparable even more potent inhibitory activity against HDAC1, HDAC6 and HDAC8 relative to the approved HDAC inhibitor SAHA. It is worth noting that although **P1** could not lead to intracellular HDAC degradation after 6 h of treatment, it could dramatically decrease the intracellular levels of HDAC1, HDAC6 and HDAC8 after 24 h of treatment. Intriguingly, the similar phenomenon was also observed in the HDAC inhibitor SAHA. Cotreatment with proteasome inhibitor bortezomib could not reverse the HDAC decreasing effects of **P1** and SAHA, confirming that their HDAC decreasing effects were not due to protein degradation. Moreover, all three bestatin-based hydroxamic acids **P1**, **P2** and **P3** exhibited more potent aminopeptidase N (APN, CD13) inhibitory activities than the approved APN inhibitor bestatin, which translated to their superior anti-angiogenic activities. Taken together, a novel bestatin-SAHA hybrid was developed, which worked as a potent APN and HDAC dual inhibitor instead of a PROTAC.

## 1. Introduction

Histone deacetylases (HDACs) can catalyze the removal of acetyl group from lysine residues of histones or some non-histone proteins, therefore playing an essential role in the regulation of gene expression and protein function [1]. To date, five HDAC inhibitors have been approved as anticancer agents [2]. Beyond cancer, HDAC inhibitors are also considered a class of potential therapeutic intervention for neurological disorders, immune diseases, cardiovascular diseases as well as age-related disorders [3,4,5].

**Figure 1:**
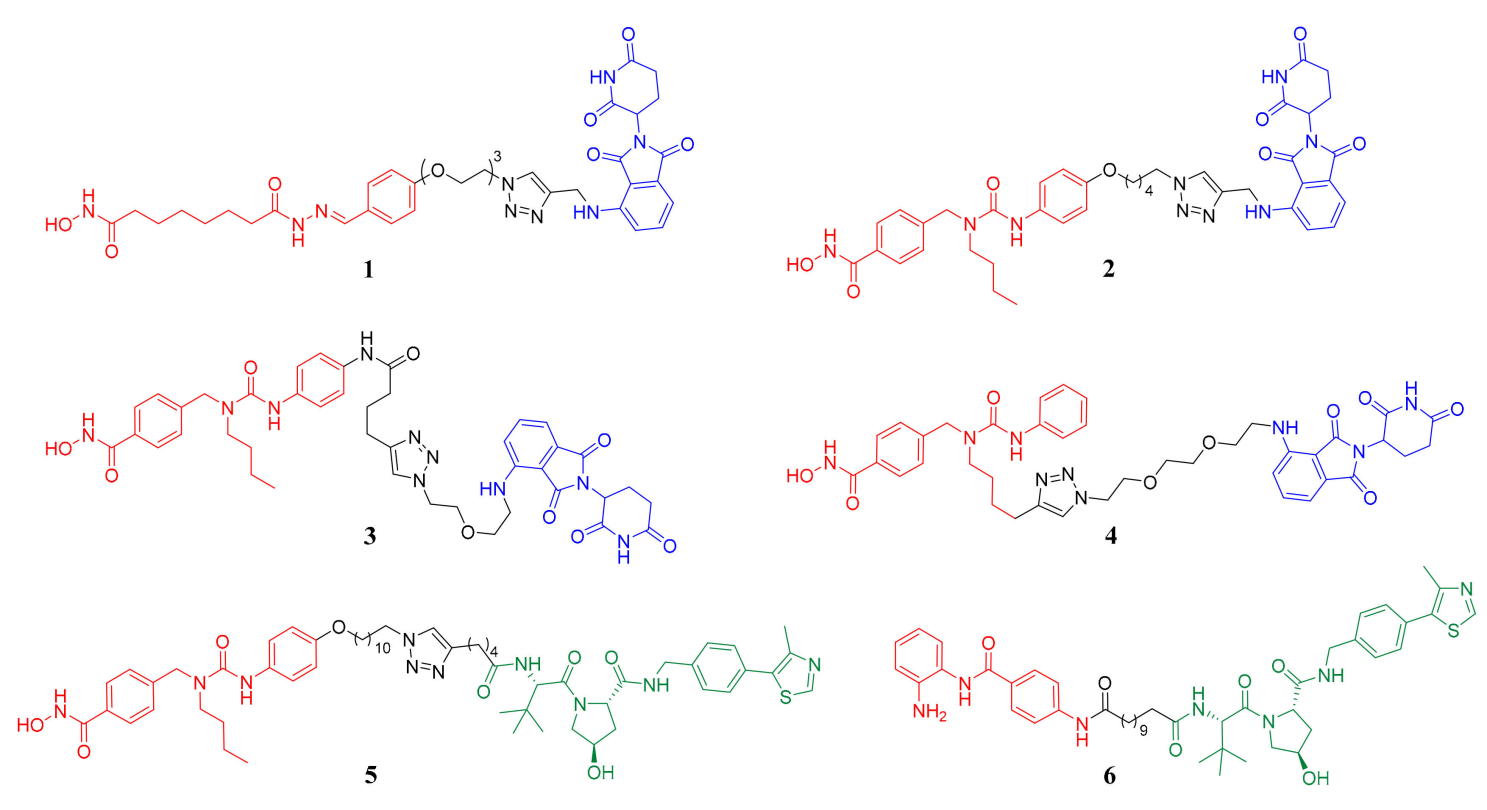
The structures of reported histone deacetylase (HDAC) degraders. The HDAC binding parts are in red, the linker parts are in black and the cereblon (CRBN) and von Hippel-Lindau (VHL) ligands are in blue and green, respectively.

An alternative strategy for inhibiting the biological function of proteins is promoting degradation of the target proteins, such as proteolysis-targeting chimeras (PROTACs), a newly developed technique to specifically knockdown the proteins of interest using the cellular ubiquitin-proteasome system [6,7,8,9]. In the aspect of chemical structure, PROTACs are generally bifunctional molecules consisting of three parts: a warhead that binds the protein of interest, an E3-ligase recruiting moiety and a linker that connects the above two parts. In the aspect of biological function, PROTACs can bring the protein of interest and E3-ligase to be adjacent to each other, then E3-ligase facilitates the transfer of ubiquitin to the target protein, consequently leading to the degradation of the ubiquitinated target protein by proteasome [6,7,8,9]. Thus far, the most widely used E3 ligases are cereblon (CRBN) and von Hippel-Lindau (VHL) [8,9]. In the research field of PROTACs for HDAC degradation, several effective degraders recruiting CRBN (compounds **1**, **2**, **3** and **4**) or VHL (compounds **5** and **6**) have already been successfully developed, among which PROTACs **1**–**5** are all HDAC6 degraders, while PROTAC **6** can effectively degrade HDAC1, HDAC2 and HDAC3 (Figure 1) [10,11,12,13,14,15].

**Figure 2:**
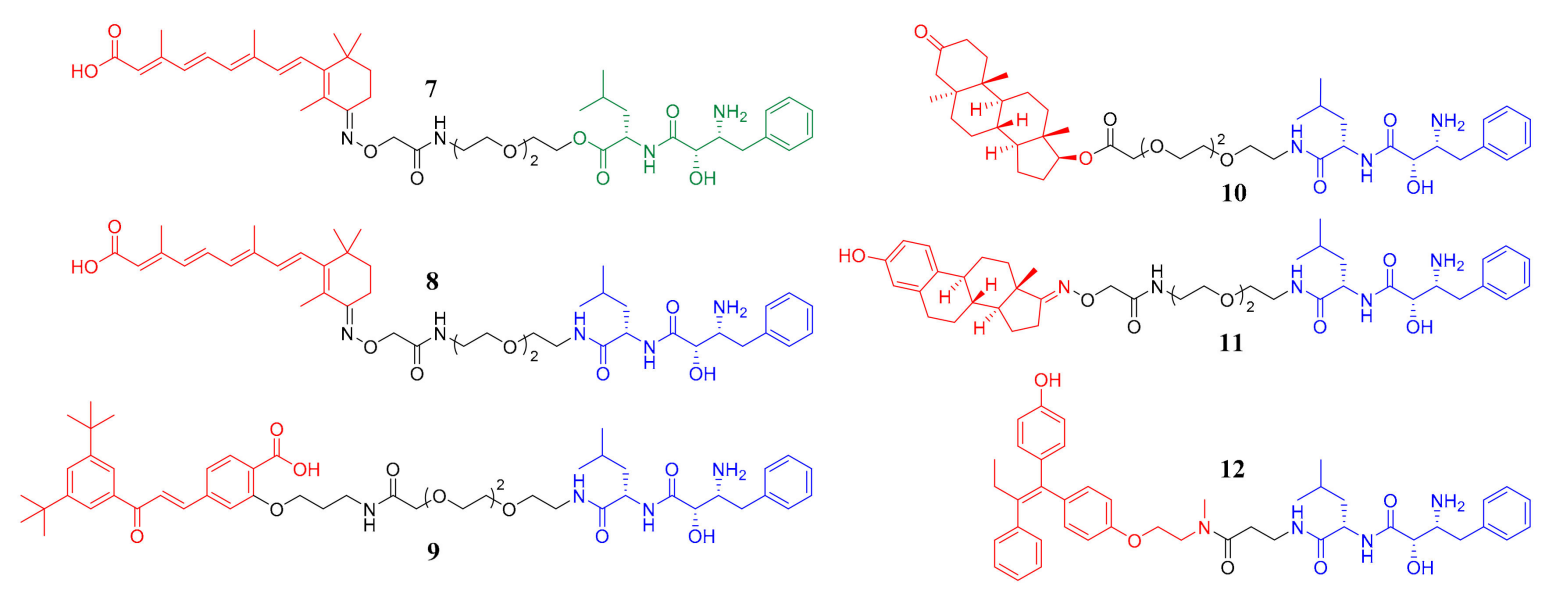
The structures of reported bestatin-based SNIPERs. The target protein binding parts are in red, the linker parts are in black and the bestatin ester and bestatin amide are in green and blue, respectively.

Besides CRBN and VHL, cellular inhibitor of apoptosis protein 1 (cIAP1) is another important E3 ligase used in the design of PROTACs [16,17,18,19,20,21,22]. The PROTACs recruiting the IAPs E3 ligases were also named SNIPERs, which was the abbreviation of specific and nongenetic IAPs-dependent protein eraser [17]. Bestatin ester and bestatin amide were the most extensively investigated cIAP1 ligands in the design of SNIPERs as shown in Figure 2. To be specific, compounds **7** and **8** are both cellular retinoic acid-binding proteins (CRABPs) degraders recruiting cIAP1 [16,17]. Compounds **9**, **10** and **11** can lead to the degradation of retinoic acid receptor *α* (RAR*α*), androgen receptor (AR) and estrogen receptor *α* (ER*α*), respectively [18]. Compound **12** is also an ER*α* degrader [19]. However, to the best of our knowledge, no HDAC degrader recruiting cIAP1 has been reported to date.

**Figure 3:**
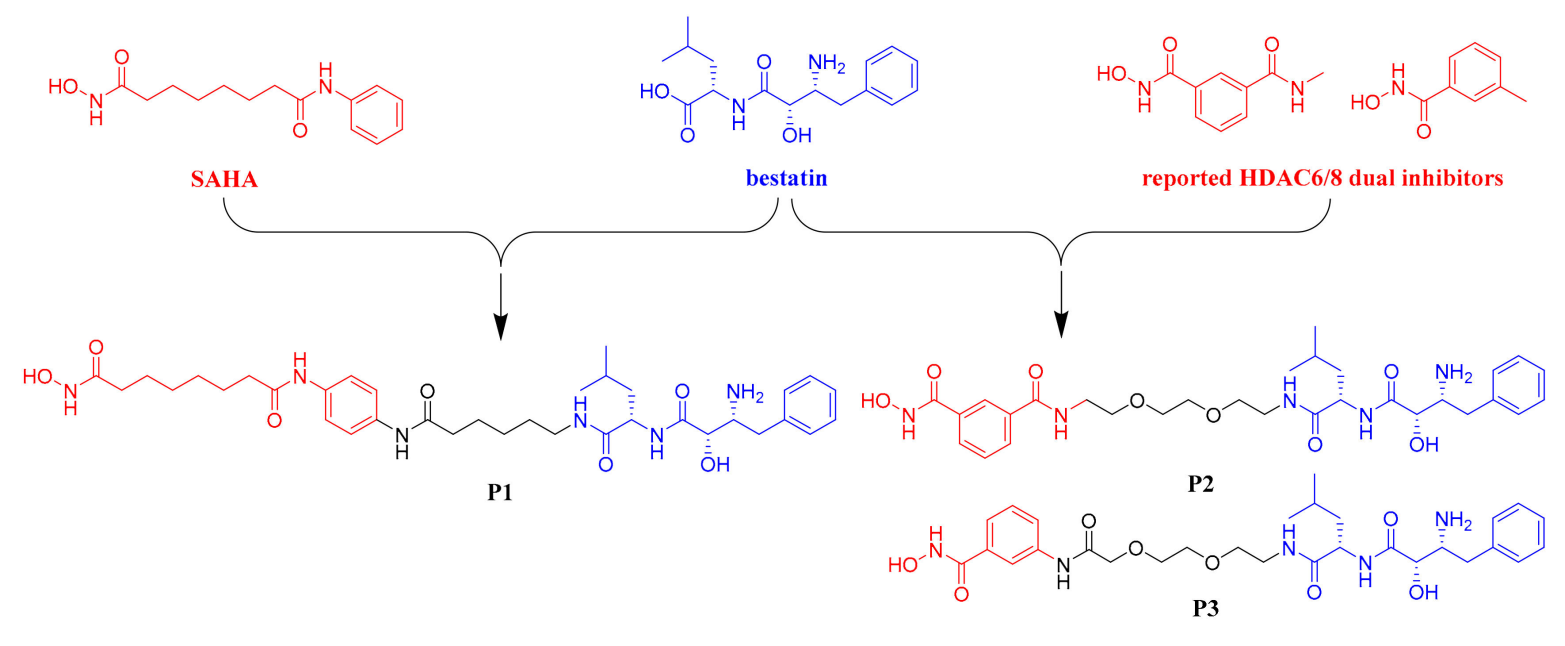
Design of bestatin-based hydroxamic acids.

To develop HDAC degrader recruiting cIAP1, we herein designed and synthesized three bestatin-based hydroxamic acids (**P1**, **P2** and **P3**). As shown in Figure 3, compound **P1** was a hybrid molecule of bestatin and the approved pan-HDAC inhibitor SAHA. Compounds **P2** and **P3** were designed based on the structural feature of two reported HDAC6/8 dual inhibitors with very low molecular weights [23]. All three target compounds contained the bestatin amide scaffold as the cIAP1 recruiting ligand and the hydroxamic acid-based warhead to target HDAC. Biological studies revealed that although the bestatin-SAHA hybrid **P1** possessed potent inhibitory activities against HDAC1, HDAC6 and HDAC8, none of the three HDAC isoforms could be degraded by **P1** after 6 h of treatment, probably due to the inappropriate length of the linker connecting bestatin and SAHA in **P1**. Intriguingly, both **P1** and SAHA could lead to a proteasome-independent decrease of the intracellular levels of HDAC1, HDAC6 and HDAC8 after 24 h of treatment. Another interesting result was that **P1**, **P2** and **P3** all exhibited potent aminopeptidase N (APN) inhibitory activities and anti-angiogenic activities, which were even better than those of the approved APN inhibitor bestatin.

## 2. Results and Discussion

### 2.1. Chemistry

**Scheme 1:**
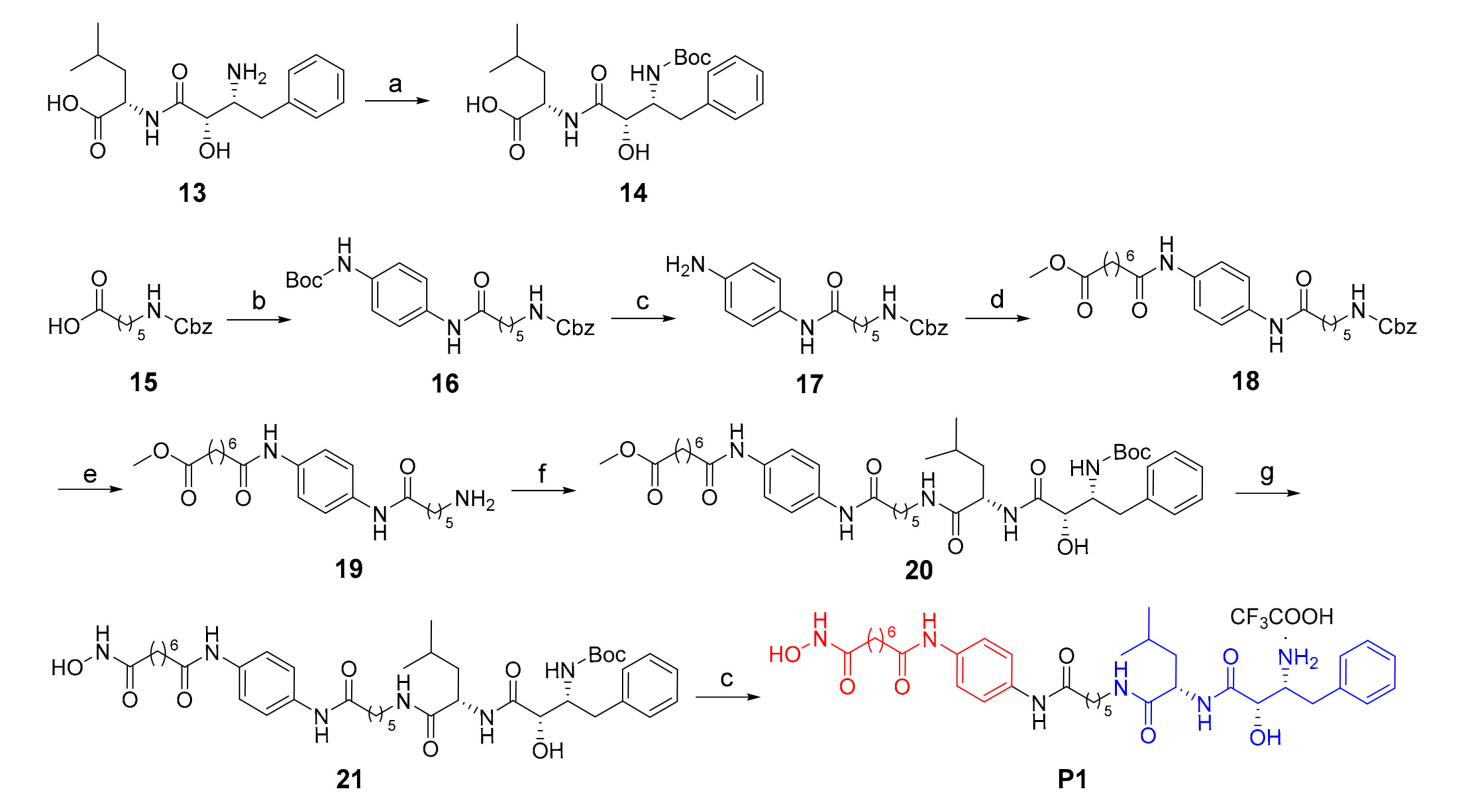
Reagents and conditions: (**a**) (Boc)_2_O, DIPEA, DCM, 83%; (**b**) *N*-Boc-*p*-phenylenediamine, EDCI, HOBT, DCM, 85%; (**c**) CF_3_COOH, DCM, 95% for **17**, 32% for **P1**; (**d**) suberic acid monomethyl ester, EDCI, HOBT, DMF, 80 °C, 62%; (**e**) 10% Pd/C, ammonium formate, MeOH, THF, 65 °C, 92%; (**f**) **14,** EDCI, HOBT, DMF, 80 °C, 58%; (**g**) NH_2_OK/MeOH, 65%.

The synthesis of target compound **P1** was described in Scheme 1. Boc-protection of the starting material **13** led to compound **14**. Condensation of **15** with *N*-Boc-*p*-phenylenediamine using the coupling reagent EDCI/HOBT led to **16.** Then, Boc-deprotection in acidic condition gave intermediate **17**, which was condensed with suberic acid monomethyl ester to afford compound **18**. The deprotection of compound **18** was conducted using ammonium formate as reductive reagent, given to compound **19**, which coupled with compound **14** to afford intermediate **20**. The methyl ester group of **20** was transformed into hydroxamate moiety with freshly prepared NH_2_OK to give compound **21**. Finally, Boc-deprotection in the presence of CF_3_COOH gave the target product **P1**.

**Scheme 2:**
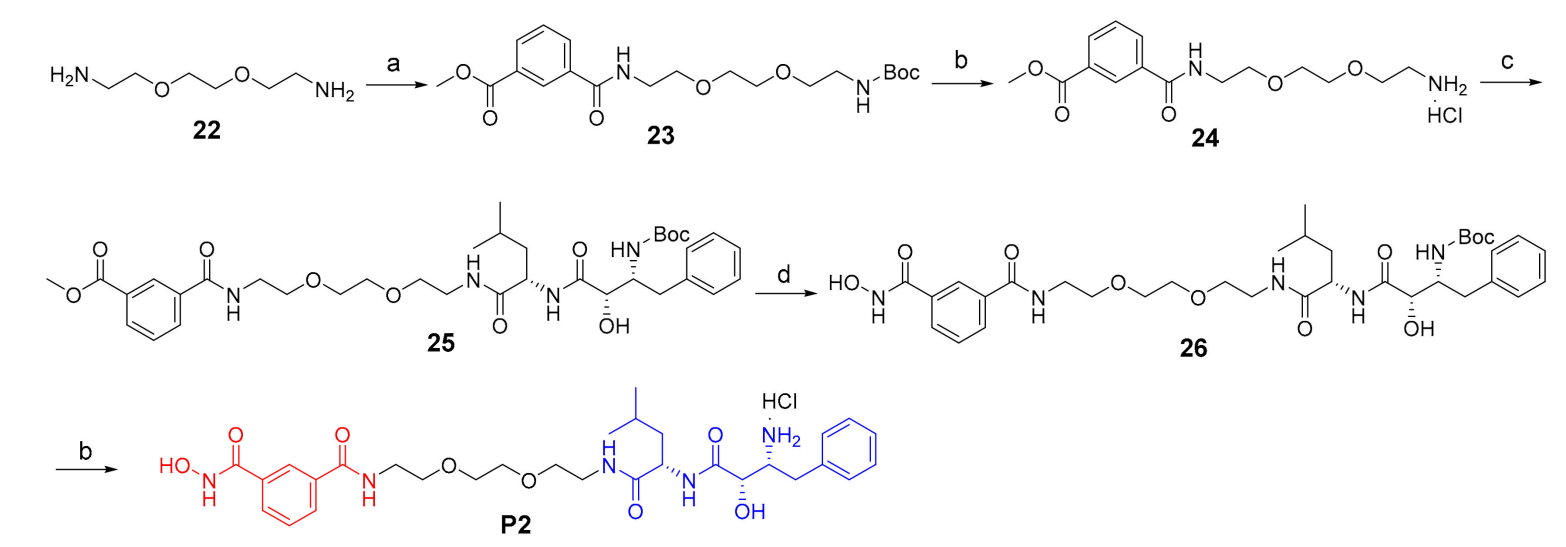
Reagents and conditions: (**a**) 1) (Boc)_2_O, DIPEA, DCM, 66%; 2) 3-(methoxycarbonyl)benzoic acid, HATU, DIPEA, DMF, 88%; (**b**) HCl/dioxane, 11% for **P2**; (**c**) **14,** HATU, DIPEA, DMF, 82%; (**d**) NH_2_OK/MeOH.

The synthesis of target compound **P2** was illustrated in Scheme 2. Firstly, Boc-protection of compound **22**, followed by condensation with 3-(methoxycarbonyl)benzoic acid, led to compound **23**. Boc-deprotection of compound **23** using HCl/dioxane led to compound **24**. After that, compound **24** coupled with compound **14** to give intermediate **25**. Then compound **25** reacted with freshly prepared NH_2_OK to give compound **26**. Finally, Boc-deprotection using HCl/dioxane afforded the target product **P2**.

**Scheme 3:**
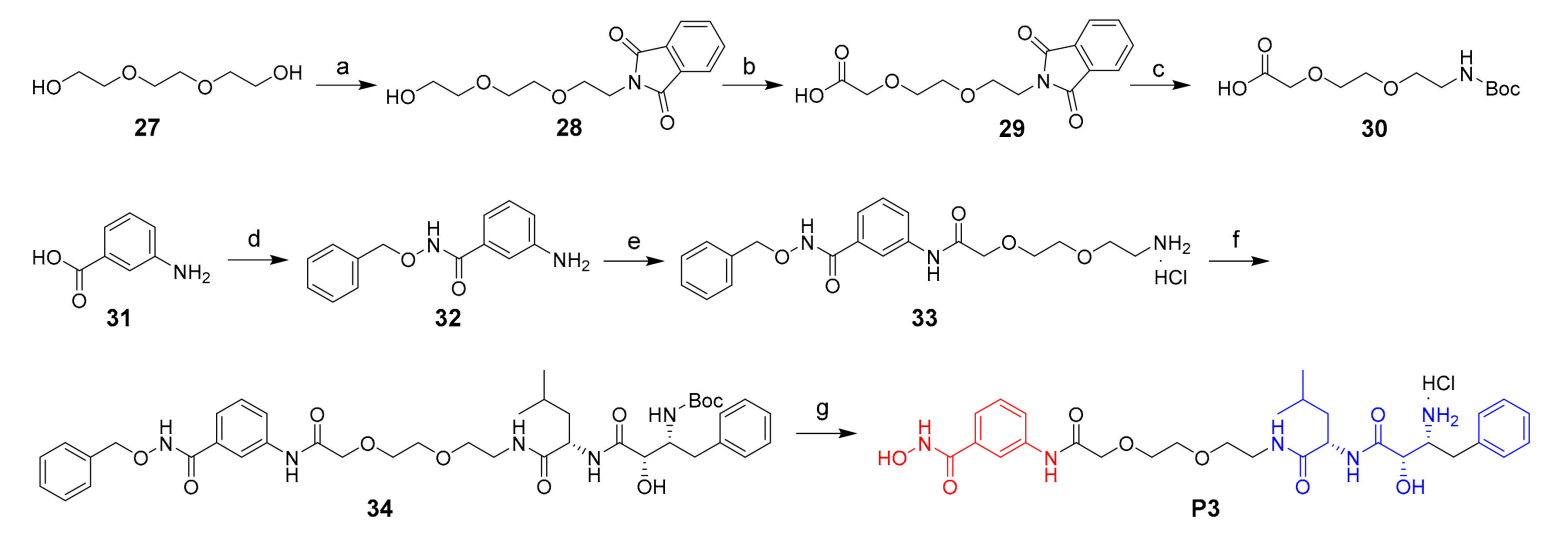
Reagents and conditions: (**a**) isoindoline-1,3-dione, PPh_3_, DIAD, THF, 57%; (**b**) PhI(AcO)_2_, TEMPO, CN/H_2_O, 70 °C; (**c**) 1) N_2_H_4_^.^H_2_O, EtOH, 80 °C; 2) (Boc)_2_O, NaOH, THF; yield: 41% for two steps; (**d**) *O*-benzylhydroxylamine hydrochloride, HATU, DIPEA, DMF, 71%; (**e**) 1) **30,** HATU, DIPEA, DMF; 2) HCl/dioxane; (**f**) **14,** HATU, DIPEA, DMF, 50%; (**g**) 1) 10% Pd/C, H_2_, MeOH; 2) HCl/dioxane; yield: 8% for two steps.

The synthesis of target compound **P3** was described in Scheme 3. The starting compound **27** went through Mitsunobu reaction to give compound **28**, which then was oxidized to give the carboxylic acid derivatives **29**. Then, compound **29** went through hydrazinolysis and Boc-protection to give compound **30**. 2-amino benzoic acid **31** coupled with *O*-benzylhydroxylamine hydrochloride gave compound **32**, which went through condensation, Boc-deprotection and condensation to give compound **34**. Finally, the Cbz and Boc groups were deprotected to give the target product **P3**.

### 2.2. In Vitro HDACs Inhibitory Assay

Based on our compound design strategy, we believed that compound **P1** could retain the potent pan-HDAC inhibitory activity of SAHA and hoped that **P2** and **P3** could exhibit HDAC6 and HDAC8 selective profiles. The HDAC inhibitory potency of **P1**, **P2** and **P3** was preliminarily evaluated by determining their inhibitory rates against HDAC1/6/8 at a concentration of 10 μM. The results in Table 1 showed that **P1** exhibited promising pan-HDAC inhibitory potency with inhibitory rates of over 90% against all three tested HDAC isoforms. However, the HDAC inhibitory rates of compounds **P2** and **P3** were relatively low, and no obvious isoform selectivity was observed. According to the preliminary results, only **P1** was progressed to the determination of IC_50_. As shown in Table 1, the IC_50_ values of **P1** against HDAC1/6/8 were all lower than those of the positive control SAHA, confirming the potent HDAC inhibitory activity of **P1**, especially against HDAC1 (IC_50_ = 30.0 ± 2.8 nM) and HDAC6 (IC_50_ = 11.5 ± 0.71 nM). The higher potency of **P1** compared to SAHA could just come from the capping effect brought by the additional bestatin moiety.

### 2.3. Western Blot Analysis

**Figure 4:**
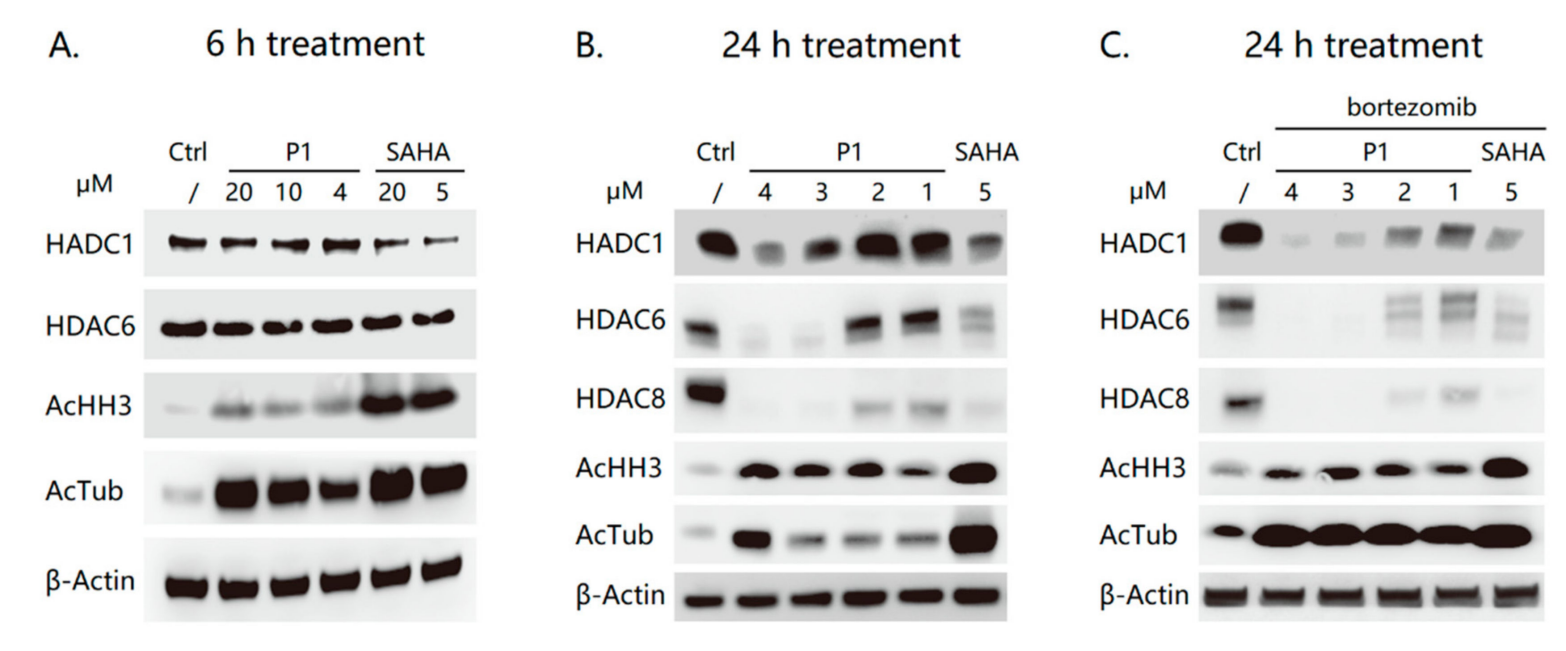
Western blot analysis of levels of proteins of interest in RPMI-8226 cells. (**A**) The cells were treated with **P1** and SAHA at the indicated concentrations for 6 h. (**B**) The cells were treated with **P1** and SAHA at the indicated concentrations for 24 h. (**C**) The cells were pre-incubated with 10 nM bortezomib before the treatment of **P1** and SAHA at the indicated concentrations for 24 h.

Considering its potent HDAC1 and HDAC6 inhibitory activity, compound **P1** was further evaluated by western blot analysis to see if it could promote the degradation of HDAC1 and HDAC6 in human multiple myeloma RPMI-8226 cells. As shown in Figure 4A, compound **P1** did not lead to HDAC1 or HDAC6 depletion at the concentration up to 20 μM after a 6-hour treatment, though it showed effective intracellular target engagement evidenced by the increased levels of acetyl-histone H3 (the HDAC1 substrate) and acetyl-*α*-tubulin (the HDAC6 substrate). As expected, the HDAC inhibitor SAHA potently inhibited the intracellular HDAC without influencing the levels of HDAC1 and HDAC6 after a 6-hour treatment (Figure 4A). It was worth noting that 24-hour treatment of **P1** at concentrations ranging from 1 μM to 4 μM could significantly decrease the levels of HDAC1, HDAC6 and even HDAC8 in a dose-dependent manner (Figure 4B). More surprisingly, 5 μM SAHA could also effectively lead to the downregulation of HDAC1, HDAC6 and HDAC8, indicating the downregulation was not due to proteasome degradation. This was confirmed by the results that the proteasome inhibitor bortezomib could not block the HDAC downregulation as shown in Figure 4C. Our results showed evidence that some, if not all, HDAC inhibitors possess the HDAC downregulation effects in specific cell lines. Therefore, in the development of HDAC inhibitor-based PROTACs, it is quite important to check if the observed HDAC downregulation is due to the PROTAC-mediated proteasome degradation.

### 2.4. In Vitro APN Inhibitory Assay

Considering that the structures of **P1, P2** and **P3** contain the scaffold of bestatin, which is an aminopeptidase N (APN, CD13) inhibitor approved as antitumor immunomodulator [24], the in vitro APN inhibitory activities of **P1, P2** and **P3** were investigated with bestatin as the positive control. The results in Table 2 showed that all three bestatin-based compounds exhibited potent APN inhibitory activities, among which compound **P2** was the most potent one with IC_50_ value of 1.67 ± 0.03 μM. Notably, the APN inhibitory activities of **P1, P2** and **P3** were even more potent than that of bestatin, indicating that introduction of large functional groups to the carboxy of bestatin was tolerated for its APN inhibitory activity. These results bring it to our attention that APN inhibition is a potential off-target effect of bestatin-based PROTACs.

### 2.5. Ex Vivo Rat Thoracic Aorta Rings (TARs) Assay

**Figure 5:**
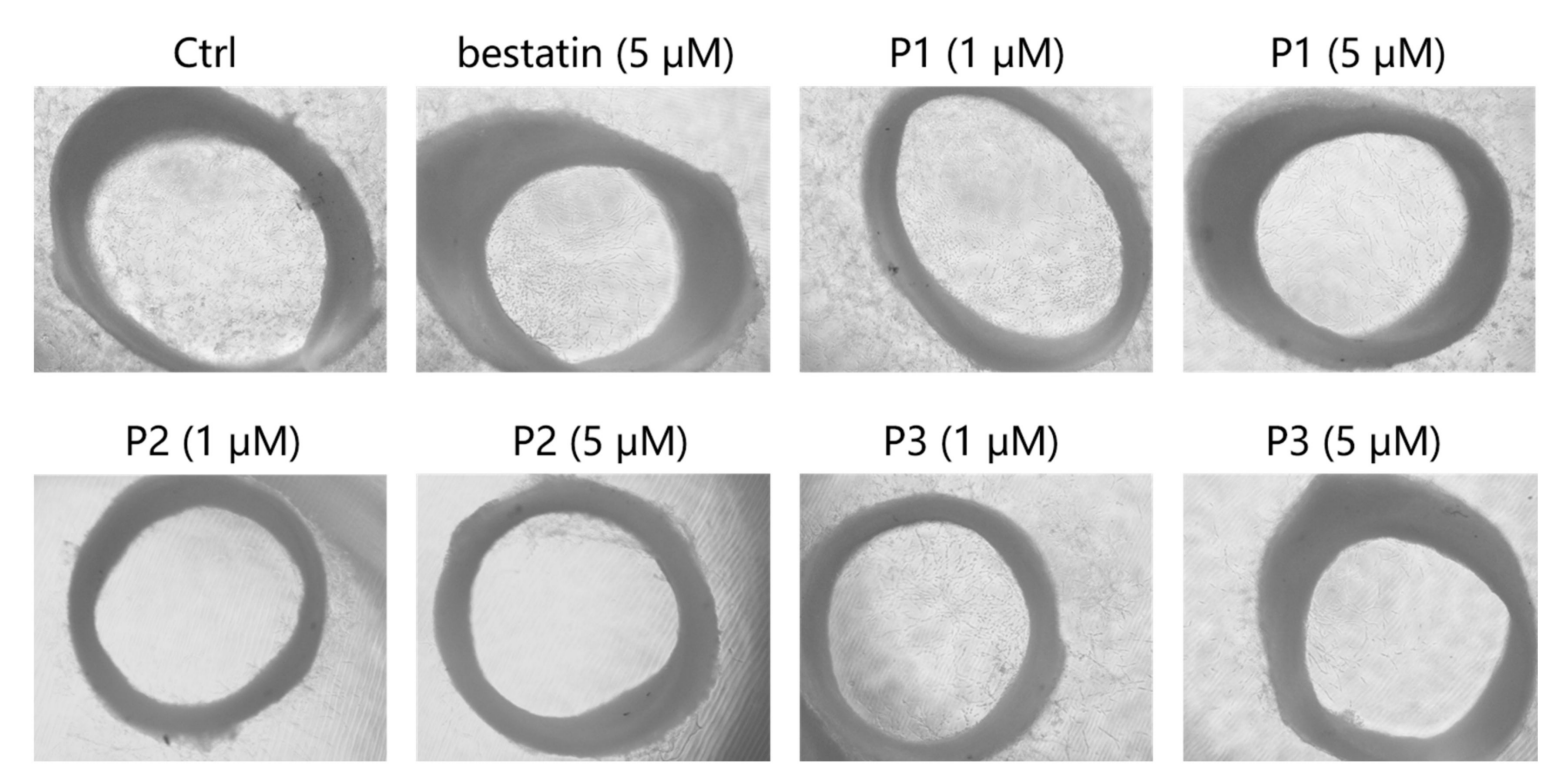
Representative images of the rat thoracic aorta rings treated with compounds at the indicated concentrations.

APN is a moonlighting metalloenzyme playing crucial roles in various functions such as angiogenesis and metastasis of tumor cells [25]. Encouraged by their remarkable APN inhibitory activities, compounds **P1, P2** and **P3** were progressed to rat thoracic aorta rings (TARs) assay to evaluate their anti-angiogenesis potencies. As presented in Figure 5, considerable microvessels sprouted from the thoracic aorta ring in the negative control group. In contrast, **P1, P2** and **P3** effectively inhibited the microvessel outgrowth in a dose-dependent manner. Consistent with the APN inhibitory activities shown in Table 2, compounds **P1, P2** and **P3** exhibited superior anti-angiogenesis activities relative to bestatin at the same concentration of 5 μM. Note that compound **P2,** which possessed the most potent APN inhibitory activity among the three analogs, could almost completely inhibit the microvessel outgrowth at the concentration of 5 μM.

## 3. Materials and Methods

### 3.1. Chemistry

All commercially available starting materials, reagents and solvents were used without further purification unless otherwise stated. All reactions were monitored by thin-layer chromatography on 0.25 mm silica gel plates (60GF-254). UV light, iodine stain and ferric chloride were used to visualize the spots. ^1^H-NMR and ^13^C-NMR spectra were recorded on a Bruker DRX spectrometer (Bruker, Billerica, MA, USA) at 400 MHz and 100 MHz, with δ given in parts per million (ppm) and *J* in hertz (Hz) and using TMS as an internal standard. High-resolution mass spectra were performed by Shandong Analysis and Test Center in Jinan, China. ESI-MS spectra were recorded on an API 4000 spectrometer (SCIEX, Framingham, MA, USA). Silica gel was used for column chromatography purification.

#### 3.1.1. The Synthesis of Target Compound **P1**

##### ((2S,3R)-3-((Tert-butoxycarbonyl)amino)-2-hydroxy-4-phenylbutanoyl)-l-leucine (**14**)

To a solution of compound **13** (0.50 g, 1.62 mmol) and DIPEA (0.63 g, 4.86 mmol) in anhydrous DCM (10 mL) was added (Boc)_2_O (0.43 g, 1.95 mmol) at 20 °C, the reaction mixture was allowed to stir for 12 h. Then, the mixture was concentrated in vacuum to give residue, which was purified by silica gel chromatography to give 0.55 g white solid, yield: 83%. ^1^H-NMR (400 MHz, DMSO-*d*_6_): δ 12.69 (s, 1H), 7.76 (d, *J* = 8.0 Hz, 1H), 7.29–7.15 (m, 5H), 6.14 (d, *J* = 12.0 Hz, 1H), 6.04 (s, 1H), 4.33–4.27 (m, 1H), 3.95–3.83 (m, 2H), 2.82–2.77 (m, 1H), 2.67–2.62 (m, 1H), 1.67–1.58 (m, 2H), 1.51–1.46 (m, 1H), 1.29–1.15 (m, 9H), 0.88–0.80 (m, 6H).

##### Tert-butyl (4-(6-(((benzyloxy)carbonyl)amino)hexanamido)phenyl)carbamate (**16**)

EDCI (2.11 g, 11.00 mmol) and HOBT (1.49 g, 11.00 mmol) were added into the solution of compound **15** (2.92 g, 11.00 mmol) in anhydrous DCM (50 mL) at 0 °C. The mixture was stirred at 0 °C for 30 min and followed by adding *N*-Boc-*p*-phenylenediamine (2.08 g, 10.00 mmol). The mixture was stirred at 0 °C for another 10 min and then stirred at 25 °C for 12 h. Filtration and the residue was washed with DCM to give 3.87 g of compound **16** as a white solid, yield: 85%. ^1^H-NMR (400 MHz, DMSO-*d*_6_): δ 9.65 (s, 1H), 9.14 (s, 1H), 7.37–7.35 (d, *J* = 8.0 Hz, 2H), 7.29–7.15 (m, 7H), 4.91 (s, 2H), 2.92–2.88 (m, 2H), 2.18–2.14 (m, 2H), 1.50–1.44 (m, 2H), 1.38–1.30 (m, 11H), 1.21–1.17 (m, 2H).

##### Benzyl (6-((4-aminophenyl)amino)-6-oxohexyl)carbamate (**17**)

Compound **16** (2.28 g, 5.00 mmol) was dissolved in DCM (5 mL) and trifluoroacetic acid (10 mL) at 0 °C. The mixture was stirred at 25 °C for 2 h. The solvent was evaporated in vacuum and the residue was re-dissolved in water (50 mL). The pH was adjusted to 6 with saturated NaHCO_3_. The precipitate was filtered and dried in vacuum to give 1.69 g white solid, yield: 95%. ^1^H-NMR (400 MHz, DMSO-*d*_6_): δ 9.42 (s, 1H), 7.38–7.25 (m, 5H), 7.20 (d, *J* = 8.0 Hz, 2H), 6.47 (d, *J* = 8.0 Hz, 2H), 5.00 (s, 2H), 4.82 (s, 2H), 3.00–2.96 (m, 2H), 2.21–2.17 (m, 2H), 1.56–1.50 (m, 2H), 1.43–1.40 (m, 2H), 1.28–1.24 (m, 2H).

##### Methyl 8-((4-(6-(((benzyloxy)carbonyl)amino)hexanamido)phenyl)amino)-8-oxooctanoate (**18**)

EDCI (2.11 g, 11.00 mmol) and HOBT (1.49 g, 11.00 mmol) were added into the solution of suberic acid monomethyl ester (2.07 g, 11.00 mmol) in DMF (20 mL) at 0 °C. The mixture was stirred at 0 °C for 30 min and followed by adding compound **17** (3.55 g, 10.00 mmol). The mixture was stirred at 0 °C for 10 min and then stirred at 80 °C for 7 h. The mixture was poured into water (100 mL). The formed precipitate was filtered off and washed with DCM (20 mL), then dried in vacuum to give 3.26 g white solid, yield: 62%. ^1^H-NMR (400 MHz, DMSO-*d*_6_): δ 9.78 (s, 2H), 7.73–7.67 (m, 1H), 7.48 (s, 4H), 7.38–7.24 (m, 5H), 5.00 (s, 2H), 3.58 (s, 3H), 3.01–2.96 (m, 2H), 2.31–2.24 (m, 6H), 1.58–1.49 (m, 6H), 1.46–1.39 (m, 2H), 1.30–1.28 (m, 4H), 0.89–0.84 (m, 2H).

##### Methyl 8-((4-(6-aminohexanamido)phenyl)amino)-8-oxooctanoate (**19**)

Compound **18** (1.05 g, 2.00 mmol) was dissolved in THF (10 mL) and MeOH (10 mL) and followed by the addition of ammonium formate (0.50 g, 8.00 mmol) and 10% Pd/C (0.10 g). The mixture was stirred at 65 °C for 3 h. Then Pd/C was filtered out and the solvent was evaporated in vacuum to give 0.72 g white solid, yield: 92%. ^1^H-NMR (400 MHz, DMSO-*d*_6_): δ 9.78 (d, *J* = 3.6 Hz, 2H), 7.48 (s, 4H), 3.58 (s, 3H), 2.54–2.51 (m, 2H), 2.31–2.24 (m, 6H), 1.60–1.50 (m, 6H), 1.39–1.24 (m, 8H).

##### Methyl 8-((4-((6*R*,7*S*,10*S*)-6-benzyl-7-hydroxy-10-isobutyl-2,2-dimethyl-4,8,11-trioxo-3-oxa-5,9,12-triazaoctadecan-18-amido)phenyl)amino)-8-oxooctanoate (**20**)

EDCI (0.42 g, 2.20 mmol) and HOBT (0.30 g, 2.20 mmol) were added into the solution of compound **14** (0.86 g, 2.10 mmol) in DMF (20 mL) at 0 °C. The mixture was stirred at 0 °C for 30 min and followed by the addition of compound **19** (0.78 g, 2.00 mmol). The mixture was stirred at 0 °C for another 10 min and then stirred at 80 °C for 7 h. The mixture was poured into water (100 mL). The formed precipitate was filtered off to afford the crude product, which was purified by column chromatography (DCM/MeOH = 20:1). The obtained product was further recrystallized from ethyl acetate/petroleum ether (1:1) to give 0.91 g white solid, yield: 58%. ^1^H-NMR (400 MHz, DMSO-*d*_6_): δ 9.78 (s, 2H), 8.01–7.98 (m, 1H), 7.59 (d, *J* = 8.0 Hz, 1H), 7.48 (s, 4H), 7.27–7.18 (m, 5H), 6.20 (d, *J* = 8.0 Hz, 1H), 5.96 (d, *J* = 8.0 Hz, 1H), 4.34–4.28 (m, 1H),3.98–3.92 (m, 1H), 3.82–3.80 (m, 1H), 3.58 (s, 3H), 3.04–3.01 (m, 2H), 2.81–2.76 (m, 1H), 2.69–2.64 (m, 1H), 2.31–2.24 (m, 6H), 1.58–1.51 (m, 6H), 1.44–1.39 (m, 2H), 1.30–1.15 (m, 18H), 0.86–0.81 (m, 6H).

##### Tert-butyl ((2*R*,3*S*)-3-hydroxy-4-(((*S*)-1-((6-((4-(8-(hydroxyamino)-8-oxo-octanamido)phenyl) amino)-6-oxohexyl)amino)-4-methyl-1-oxopentan-2-yl)amino) -4-oxo-1-phenylbutan-2-yl)carbamate (**21**)

Compound **20** (0.78 g, 1.00 mmol) was added into the freshly prepared potassium hydroxylamine methanol solution (5 mL), and the mixture was stirred at 25 °C for 0.5 h. The mixture was poured into water (30 mL) and was acidified with 5% citric acid to pH = 6. The formed precipitate was filtered and dried to give 0.51 g white solid, yield: 65%. ^1^H-NMR (400 MHz, DMSO-*d*_6_): δ 10.34 (s, 1H), 9.78 (s, 2H), 8.67 (s, 1H), 7.99 (t, *J* = 4.0 Hz, 1H), 7.59 (d, *J* = 8.0 Hz, 1H), 7.48 (s, 4H), 7.27–7.18 (m, 5H), 6.20 (d, *J* = 8.0 Hz, 1H), 5.98 (d, *J* = 8.0 Hz, 1H), 4.34–4.28 (m, 1H), 3.98–3.92 (m, 1H), 3.80 (s, 1H), 3.07–2.99 (m, 2H), 2.81–2.76 (m, 1H), 2.69–2.63 (m, 1H), 2.27–2.23 (m, 4H), 1.93 (t, *J* = 8.0 Hz, 2H), 1.59–1.36 (m, 8H), 1.28–1.15 (m, 18H), 0.86–0.79 (m, 6H).

##### *N*^1^-(4-(6-((*S*)-2-((2*S*,3*R*)-3-Amino-2-hydroxy-4-phenylbutanamido)-4-methylpentanamido) hexanamido)phenyl)-*N*^8^-hydroxyoctanediamide trifluoroacetate (**P1**)

Compound **21** (0.39 g, 0.50 mmol) was dissolved in DCM (2 mL) and CF_3_COOH (2 mL). The mixture was stirred at 25 °C for 1 h. The solvent was evaporated and the residue was added EtOAc. The formed precipitate was filtered and dried to give the desired product **P1** (0.13 g, yield: 32%) as white solid (CF_3_COOH salt, purity: 95.2%). ^1^H-NMR (400 MHz, DMSO-*d*_6_): δ 10.34 (s, 1H), 9.79 (s, 2H), 8.67 (s, 1H), 8.09 (t, *J* = 8.0 Hz, 1H), 8.01 (d, *J* = 8.0 Hz, 1H), 7.89 (s, 3H), 7.48 (s, 4H), 7.37–7.26 (m, 5H), 6.60 (d, *J* = 4.0 Hz, 1H), 4.28–4.23 (m, 1H), 4.00–3.97 (m, 1H), 3.54 (s, 1H), 3.09–2.93 (m, 2H), 2.82–2.63 (m, 1H), 2.27–2.23 (m, 4H), 1.93 (t, *J* = 8.0 Hz, 2H), 1.57–1.40 (m, 10H), 1.29–1.23 (m, 7H), 0.89–0.86 (m, 6H). ^13^C-NMR (100 MHz, DMSO-*d*_6_): δ 171.85 (C=O), 171.35 (C=O), 171.26 (C=O), 170.98 (C=O), 169.57 (C=O), 136.64 (ArC), 135.06 (ArC), 129.88 (ArC), 129.10 (ArC), 127.41 (ArC), 119.83 (ArC), 68.66, 54.74, 51.73, 41.56, 38.95, 36.73, 36.67, 35.22, 32.70, 29.23, 28.90, 28.87, 26.51, 25.54, 25.51, 25.30, 24.72, 23.25, 22.44.

#### 3.1.2. The Synthesis of Target Compound **P2**

##### Methyl 3-((2,2-dimethyl-4-oxo-3,8,11-trioxa-5-azatridecan-13-yl)carbamoyl)-benzoate (**23**)

To a solution of compound **22** (1.00 g, 6.76 mmol) and DIPEA (2.61 g, 20.27 mmol) in anhydrous DCM (20 mL) was added (Boc)_2_O (1.47 g, 6.76 mmol) in portions at 20 °C over 15 min. The reaction mixture was stirred at 20 °C for 12 h. After that, the mixture was concentrated in vacuum to give residue, which was purified by silica gel chromatography to give the key intermediate (1.10 g, yield: 66%). To a solution of 3-(methoxycarbonyl)benzoic acid (0.80 g, 4.43 mmol) in anhydrous DMF (10 mL) was added HATU (1.68 g, 4.43 mmol) and DIPEA (1.71 g, 13.29 mmol) in portions at 20 °C. The reaction mixture was stirred at 20 °C for 10 min, then the key intermediate (1.10 g, 4.43 mmol) was added and stirred at 20 °C for another 2 h. The reaction mixture was poured into water (25 mL). The aqueous layer was extrated with EtOAc (50 mL × 3), dried over sodium sulfate and concentrated in vacuum to give residue. The residue was purified by silica gel chromatography to give desired compound **23** (1.60 g, yield: 88%). ESI-MS, *m*/*z* = 411.4 [M + H]^+^.

##### Methyl 3-((2-(2-(2-aminoethoxy)ethoxy)ethyl)carbamoyl)benzoate hydrochloride (**24**)

To a solution of compound **23** (1.60 g, 3.90 mmol) in anhydrous dioxane (10 mL) was added HCl/dioxane (20 mL, 4 M) in portions at 20 °C. The reaction mixture was stirred at 20 °C for 2 h, followed by the concentrationin vacuum to give 1.35 g crude product as HCl salt, which was used for next step without further purification. ESI-MS, *m*/*z* = 311.0 [M + H]^+^.

##### Methyl 3-(((6*R*,7*S*,10*S*)-6-benzyl-7-hydroxy-10-isobutyl-2,2-dimethyl-4,8,11-trioxo-3,15,18-trioxa-5,9,12-triazaicosan-20-yl)carbamoyl)benzoate (**25**)

To a solution of compound **14** (0.50 g, 1.23 mmol) in anhydrous DMF (7 mL), HATU (0.47 g, 1.23 mmol) and DIPEA (0.48 g, 3.69 mmol) were added in portions at 20 °C. The reaction mixture was stirred at 20 °C for 10 min, then compound **24** (0.43 g, 1.23 mmol) was added and stirred at 20 °C for 2 h. The reaction mixture was poured into water (25 mL). The aqueous layer was extrated with EtOAc (50 mL × 3), dried over sodium sulfate, and concentrated in vacuum to give residue, which was purified by silica gel chromatography to afford the desired product **25** (0.71 g, 82% yield). ESI-MS, *m*/*z* = 701.4 [M + H]^+^.

##### Tert-butyl ((13*S*,16*S*,17*R*)-16-hydroxy-1-(3-(hydroxycarbamoyl)phenyl)-13-isobutyl-1,12,15-trioxo-18-phenyl-5,8-dioxa-2,11,14-triazaoctadecan-17-yl)carbamate (**26**)

Using the similar synthetic method of compound **21**, compound **25** gave compound **26** (0.51 g, crude). The residue was used for the next step reaction without purification. ESI-MS, *m*/*z* = 602.3 [M-Boc + H]^+^.

##### *N*^1^-((11*S*,14*S*,15*R*)-15-Amino-14-hydroxy-11-isobutyl-10,13-dioxo-16-phenyl-3,6-dioxa-9,12-diazahexadecyl)-*N*^3^-hydroxyisophthalamide trifluoroacetate (**P2**)

To a solution of compound **26** (0.51 g, crude) in anhydrous dioxane (10 mL), HCl/dioxane was added (20 mL, 4M) in portions at 20 °C. After being stirred at 20 °C for 2 h, the reaction mixture was concentrated in vacuum to give residue, which was purified by Pre-HPLC (gradient elution with acetonitrile/water containing 0.225% CF_3_COOH) to afford the desired product **P2** (51 mg, yield: 11%) as white solid (CF_3_COOH salt, purity: 97.4%). ^1^H-NMR (400 MHz, DMSO-*d*_6_): δ 11.32 (s, 1H), 9.14 (s, 1H), 8.66 (t, *J* = 5.6 Hz, 1H), 8.24 (d, *J* = 1.8 Hz, 1H), 8.19 (t, *J* = 5.6 Hz, 1H), 8.03 (d, *J* = 8.2 Hz, 1H), 7.96 (d, *J* = 7.8 Hz, 1H), 7.88 (dd, *J* = 12.4 Hz, *J* = 6.6 Hz, 4H), 7.54 (t, *J* = 7.7 Hz, 1H), 7.36 (dd, *J* = 8.8 Hz, *J* = 6.0 Hz, 2H), 7.32–7.24 (m, 3H), 6.64 (s, 1H), 4.32–4.26 (m, 1H), 3.98 (d, *J* = 3.3 Hz, 1H), 3.60–3.34 (m, 11H), 3.28–3.07 (m, 2H), 2.93 (dd, *J* = 13.8 Hz, *J* = 7.8 Hz, 1H), 2.80 (dd, *J* = 13.8 Hz, *J* = 6.5 Hz, 1H), 1.64–1.39 (m, 3H), 0.87 (dd, *J* = 8.0 Hz, *J* = 6.4 Hz, 6H). ^13^C-NMR (100 MHz, DMSO-*d*_6_): δ 172.20 (C=O), 171.01 (C=O), 166.26 (C=O), 164.19 (C=O), 158.95 (CF_3_COOH), 136.66 (ArC), 135.10 (ArC), 133.42 (ArC), 130.16 (ArC), 129.87 (ArC), 129.75 (ArC), 129.10 (ArC), 128.88 (ArC), 127.40 (ArC), 126.52 (ArC), 115.77 (CF_3_COOH), 70.01, 69.98, 69.30, 68.67, 54.71, 51.69, 41.53, 39.08, 35.22, 24.67, 23.27, 22.44. HRMS (AP-ESI) *m/z*, calcd. for C_30_H_44_N_5_O_8_ [M + H]^+^ 602.3190, found 602.3186.

#### 3.1.3. The Synthesis of Target Compound **P3**

##### 2-(2-(2-(2-Hydroxyethoxy)ethoxy)ethyl)isoindoline-1,3-dione (**28**)

To a solution of compound **27** (1.50 g, 10.00 mmol) and isoindoline-1,3-dione (1.47 g, 10.00 mmol) and PPh_3_ (2.62 g, 10.00 mmol) in THF (40 mL), DIAD was added (2.02 g, 10.00 mmol). After being stirred at 20 °C for 5 h, the mixture was concentrated to give a residue, which was purified by silica gel chromatography to give desired product (1.60 g, yield: 57%). ESI-MS, *m*/*z* = 280.2 [M+H]^+^.

##### 2-(2-(2-(1,3-Dioxoisoindolin-2-yl)ethoxy)ethoxy)acetic acid (**29**)

To a solution of compound **28** (1.40 g, 5.00 mmol) and PhI(AcO)_2_ (6.44 g, 20.00 mmol) in 50 mL CH_3_CN/H_2_O (*V*:*V* = 4:1) was added TEMPO (0.16 g, 1.00 mmol). The mixture was stirred at 70 °C for 5 h, then the saturated Na_2_SO_3_ aqueous (50 mL) was added. The aqueous layer was extracted with EtOAc, then the organic layer was discarded. The aqueous was adjusted pH = 3 with 2 M HCl, then extracted with EtOAc. The organic layer was dried and concentrated to give the crude (1.00 g, 3.41 mmol) used for the next step without purification. ESI-MS, *m*/*z* = 294.1 [M + H]^+^.

##### 2,2-Dimethyl-4-oxo-3,8,11-trioxa-5-azatridecan-13-oic acid (**30**)

To a solution of compound **29** (1.00 g, 3.41 mmol) in EtOH (20 mL), N_2_H_4_^.^H_2_O was added (0.50 g, 7.00 mmol). The reaction mixture was stirred at 80 °C for 5 h. The formed white solid was filtered out and the filtrate was concentrated to give a pale yellow oil. To a solution of the pale yellow oil in THF (15 mL) and 1N NaOH (7 mL) was added (Boc)_2_O (0.89 g, 4.09 mmol). The mixture was stirred at room temperature for 17 h, then water (20 mL) was added and extracted with EtOAc. The aqueous layer was adjusted pH = 4 with citric acid, then the aqueous layer was extracted with EtOAc. The mixture was dried and concentrated to give a residue, which was purified by silica gel chromatography EtOAc/PE/HCOOH (2/1/0.5% to 10/1/0.5%) to give desired compound **30** (0.37 g, yield: 41% two steps). ESI-MS, *m*/*z* = 164.4 [M-Boc + H]^+^.

##### 3-Amino-*N*-(benzyloxy)benzamide (**32**)

To a solution of compound **31** (0.37 g, 2.80 mmol) in anhydrous DMF (5 mL), HATU (1.14 g, 3.00 mmol) and DIPEA (0.77 g, 6.00 mmol) was added at 20 °C. The reaction mixture was stirred at 20 °C for 10 min, then *O*-benzylhydroxylamine hydrochloride (0.48 g, 3.00 mmol) was added. After being stirred at 20 °C for another 2 h, the reaction mixture was poured into water (25 mL). The aqueous layer was extracted with EtOAc, dried over sodium sulfate and concentrated in vacuum to give residue. The residue was purified by silica gel chromatography to give desired compound **32** (0.48 g, yield: 71%). ESI-MS, *m*/*z* = 243.3 [M + H]^+^.

##### 3-(2-(2-(2-Aminoethoxy)ethoxy)acetamido)-*N*-(benzyloxy)benzamide hydrochloride (**33**)

Using the similar synthetic method for compound **32**, compound **30** (0.37 g, 1.40 mmol) reacted with compound **32** to give a key intermediate. The solution of intermediate (0.48 g, 0.99 mmol) in HCl/dioxane (20 mL, 4 M) was allowed to stir at 20 °C for 6 h. The reaction mixture was concentrated in vacuum to give 0.40 g crude product **33** as HCl salt. ESI-MS, *m*/*z* = 388.0 [M + H]^+^.

##### Tert-butyl ((11*S*,14*S*,15*R*)-1-((3-((benzyloxy)carbamoyl)phenyl)amino)-14-hydroxy-11-isobutyl- 1,10,13-trioxo-16-phenyl-3,6-dioxa-9,12-diazahexadecan-15-yl)carbamate (**34**)

Using the similar synthetic method for compound **32**, compound **14** (0.40 g, 0.98 mmol) reacted with compound **33** to give desired product **34** (0.38 g, yield: 50%). ESI-MS, *m*/*z* = 678.5 [M-Boc + H]^+^.

##### 3-((11*S*,14*S*,15*R*)-15-Amino-14-hydroxy-11-isobutyl-10,13-dioxo-16-phenyl-3,6-dioxa-9,12- diazahexadecanamido)-*N*-hydroxybenzamide (**P3**)

To a solution of compound **34** (0.38 g, 0.56 mmol) in MeOH (10 mL) wet 10% Pd/C was added (0.10 g). The reaction mixture was stirred at 20 °C under H_2_ atmosphere for 2 h, then the Pd/C was filtered out and the mixture was concentrated to give the residue used in the next step. HCl/dioxane (5 mL, 4M) was added into the residue and stirred at 20 °C for 6 h. The reaction mixture was concentrated in vacuum and purified by Pre-HPLC (gradient elution with acetonitrile/water containing 0.225% NH_3_·H_2_O) to afford the desired product **P3** (26 mg, yield: 8%) as white solid (free base, purity: 96.1%). ^1^H-NMR (400 MHz, DMSO-*d*_6_): δ 9.83 (s, 1H), 8.30 (t, *J* = 5.6 Hz, 1H), 8.09–8.02 (m, 1H), 7.82–7.72 (m, 2H), 7.46–7.34 (m, 2H), 7.33–7.13 (m, 5H), 4.34–4.16 (m, 1H), 4.08 (s, 2H), 3.75 (dd, *J* = 12.5 Hz, *J* = 2.4 Hz, 1H), 3.67–3.51 (m, 4H), 3.41 (dt, *J* = 12.1 Hz, *J* = 6.0 Hz, 2H), 3.22–3.12 (m, 2H), 2.83–2.73 (m, 1H), 1.64–1.40 (m, 3H), 0.91–0.75 (m, 6H). HRMS (AP-ESI) *m/z*, calcd. for C_29_H_42_N_5_O_8_ [M + H]^+^ 588.3033, found 588.3032.

### 3.2. In Vitro HDACs Inhibitory Assay

All the HDAC enzymes were brought from BPS Bioscience. Ten μL of enzyme solution (HDAC1, HDAC6 or HDAC8) was mixed with different concentrations of tested compound (50 μL). The mixture was incubated at 37 °C for 5 min, followed by adding 40 μL fluorogenic substrate (Boc-Lys(acetyl)-AMC for HDAC1 and HDAC6, Boc-Lys(triflouroacetyl)-AMC for HDAC8). After incubation at 37 °C for 30 min, the mixture was quenched by addition of 100 μL of developer containing trypsin and Trichostatin A. Over another incubation at 37 °C for 20 min, fluorescence intensity was measured using a microplate reader at excitation and emission wavelengths of 390 nm and 460 nm, respectively. The inhibition rates were calculated from the fluorescence intensity readout of tested wells relative to those of control wells, and the IC_50_ values were calculated using Prism non-linear curve fitting method.

### 3.3. Western Blot Analysis

The RPMI-8226 cells were plated in flat-bottom six-well plates and allowed to grow for 12 h, and then treated with compounds or DMSO for a specific time period. Then, the cells were washed twice with cold PBS and lysed in ice-cold RIPA buffer, which comprised 50 mM Tris Base, 150 mM NaCl, 5 mM EDTA, 0.1% (*v*/*v*) SDS, 0.5% (*v*/*v*) sodium deoxycholate and 1% (*v*/*v*) Triton-x-100. Lysates were cleared by centrifugation. Protein concentrations were determined using the BCA assay. Equal amounts of cell extracts were then resolved by SDS-PAGE, transferred to nitrocellulose membrane and probed with HDAC1 antibody, HDAC6 antibody, HDAC8 antibody, acetyl-histone H3 antibody, acetyl-α-tubulin antibody and β-actin antibody, respectively. The membrane was washed twice with TBST buffer before being incubated with secondary antibodies. Images were acquired using a GE ImageQuant LAS 4000. 

### 3.4. In Vitro APN Inhibitory Assay

The inhibitory activities of target compounds against APN were determined using l-leu-*p*-nitroanilide as substrate and microsomal APN from porcine kidney microsomes (Biocol) in 50 mM PBS, pH 7.2 as enzyme. Briefly, inhibitors (40 μL), PBS (145 μL), substrate (5 μL) and APN solution (10 μL) were added into 96-well plates. The mixture was incubated at 37 °C for 30 min. The hydrolysis product *p*-nitroanilide was measured at 405 nm. The inhibition rates were calculated based on the ultraviolet absorption readout of tested wells relative to those of control wells, and the IC_50_ values were calculated using Prism non-linear curve fitting method.

### 3.5. Ex Vivo Rat Aortic Rings Assay

Matrigel (100 μL; BD biosciences, NJ) was added into test well of 96-well plates and then allowed to polymerize for 0.5 h at 37 °C. Sprague–Dawley rats that were 4 to 6 weeks old were sacrificed and aortas were harvested. Each aorta was cut into 1 mm slices and embedded in additional 100 μL of Matrigel in 96-well plates. After that, the rings were incubated for 30 min at 37 °C and 5% CO_2_. Aorta rings were treated with the vehicle (0.5% DMSO) or compounds each day for 5 days and photographed on the sixth day at ×200 magnification. All experiments involving laboratory animals were performed with the approval by the institutional guidelines of Animal Care and Use Committee at Shandong University.

## 4. Conclusions

In summary, three hydroxamic acids containing the bestatin amide scaffold were designed and synthesized. Biological evaluation revealed that the bestatin-SAHA hybrid **P1** was a potent pan-HDAC inhibitor, while the HDAC inhibitory potencies and isoform-selective profiles of **P2** and **P3** with *m*-substituted *N*-hydroxybenzamide warhead were far from satisfactory. Although **P1** exhibited potent HDAC1/6/8 inhibitory activities, it could not induce proteasome-mediated degradation of the above HDAC isoforms. It is worth noting that prolong period of treatment of **P1** or SAHA could dramatically decrease the intracellular levels of many HDAC isoforms including HDAC1/6/8 in RPMI-8226 cells through an unconfirmed mechanism. Note that this effect might be cell-type-dependent. Additionally, **P1**, **P2** and **P3** all exhibited potent APN inhibitory activities, indicating that attaching large functional group to the carboxy group of bestatin would not compromise its APN inhibitory potency. It is worth noting that a previous study reported that the combination of HDAC and aminopeptidase inhibitors is highly synergistic in multiple myeloma via the disruption of the NFκB signalling pathway [26], which indicates that our dual inhibitor **P1** might be useful as anti-multiple myeloma agent.

Though no HDAC degrader was obtained, our research drew much needed attentions for the development of PROTACs. First, pan-HDAC inhibitor SAHA is capable of downregulating the cellular levels of multi-HDAC isoforms after long period of treatment. Therefore, it is indispensable to do mechanism study to confirm that the HDAC downregulation effect of HDAC inhibitor-based PROTACs is derived from the PROTAC-mediated degradation rather than from their intrinsic HDAC inhibitory activities. Secondly, explaining the cellular effects of bestatin-based PROTACs with caution is necessary because the bestatin-based PROTACs can also inhibit APN and even other aminopeptidases.

## Figures and Tables

**Table 1:** HDAC1/6/8 inhibitory activities of compounds **P1**, **P2**, **P3** and SAHA.

Compounds	Inhibitory Rates at 10 μM (IC_50_) ^a^		
	HDAC1	HDAC6	HDAC8
**P1**	98% (30.0 ± 2.8 nM)	99% (11.5 ± 0.71 nM)	90% (694.0 ± 110.3 nM)
**P2**	66%	76%	64%
**P3**	67%	71%	51%
SAHA	63.5 ± 3.5 nM	21.0 ± 1.4 nM	>7000 nM
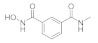	NT ^b^	2.5 ± 1.6 μM ^c^	14 ± 10 μM ^c^
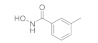	NT ^b^	0.65 ± 0.52 μM ^c^	3.3 ± 1.7 μM ^c^

^a^ Assays were performed in replicate (*n* ≥ 3). The IC_50_ values were shown as mean ± SD; ^b^ NT = not determined; ^c^ Reported in [23].

**Table 2:** APN inhibitory activities of compounds **P1**, **P2**, **P3** and bestatin.

Compounds	IC_50_ (μM) ^a^
**P1**	3.43 ± 0.14
**P2**	1.67 ± 0.03
**P3**	2.65 ± 0.16
bestatin	4.33 ± 0.50

^a^ Assays were performed in replicate (*n* ≥ 3). The data were shown as mean ± SD.

## Abbreviations

HDACs	histone deacetylases
PROTACs	proteolysis-targeting chimeras
VHL	von Hippel-Lindau
cIAP1	cellular inhibitor of apoptosis protein 1
SNIPERs	specific and nongenetic IAPs-dependent protein eraser
CRABPs	cellular retinoic acid-binding proteins
RAR*α*	retinoic acid receptor *α*
AR	androgen receptor
ER*α*	estrogen receptor *α*
APN	aminopeptidase N
TARs	rat thoracic aorta rings
ppm	parts per million
